# Clinical Deterioration during Antitubercular Treatment at a District Hospital in South Africa: The Importance of Drug Resistance and AIDS Defining Illnesses

**DOI:** 10.1371/journal.pone.0004520

**Published:** 2009-02-20

**Authors:** Dominique J. Pepper, Kevin Rebe, Chelsea Morroni, Robert J. Wilkinson, Graeme Meintjes

**Affiliations:** 1 Department of Medicine, University of Cape Town, Cape Town, South Africa; 2 Infectious Diseases Unit, GF Jooste Hospital, Manenberg, South Africa; 3 Women's Health Research Unit, School of Public Health & Family Medicine, University of Cape Town, Cape Town, South Africa; 4 Wellcome Trust Centre for Research in Clinical Tropical Medicine, Division of Medicine, Imperial College London, London, United Kingdom; 5 Institute of Infectious Diseases and Molecular Medicine, Faculty of Health Sciences, University of Cape Town, Cape Town, South Africa; 6 National Institute for Medical Research, London, United Kingdom; University of Cape Town, United Kingdom

## Abstract

**Background:**

Clinical deterioration on drug therapy for tuberculosis is a common cause of hospital admission in Africa. Potential causes for clinical deterioration in settings of high HIV-1 prevalence include drug resistant *Mycobacterium tuberculosis (M.tb)*, co-morbid illnesses, poor adherence to therapy, tuberculosis associated-immune reconstitution inflammatory syndrome (TB-IRIS) and subtherapeutic antitubercular drug levels. It is important to derive a rapid diagnostic work-up to determine the cause of clinical deterioration as well as specific management to prevent further clinical deterioration and death. We undertook this study among tuberculosis (TB) patients referred to an adult district level hospital situated in a high HIV-1 prevalence setting to determine the frequency, reasons and outcome for such clinical deterioration.

**Method:**

A prospective observational study conducted during the first quarter of 2007. We defined clinical deterioration as clinical worsening or failure to stabilise after 14 or more days of antitubercular treatment, resulting in hospital referral. We collected data on tuberculosis diagnosis and treatment, HIV-1 status and antiretroviral treatment, and investigated reasons for clinical deterioration as well as outcome.

**Results:**

During this period, 352 TB patients met inclusion criteria; 296 were admitted to hospital accounting for 17% of total medical admissions (n = 1755). Eighty three percent of TB patients (291/352) were known to be HIV-1 co-infected with a median CD4 count of 89cells/mm^3^ (IQR 38–157). Mortality among TB patients admitted to hospital was 16% (n = 48). The median duration of hospital admission was 9.5 days (IQR 4–18), longer than routine in this setting (4 days). Among patients in whom HIV-1 status was known (n = 324), 72% of TB patients (n = 232) had an additional illness to tuberculosis; new AIDS defining illnesses (n = 80) were the most frequent additional illnesses (n = 208) in HIV-1 co-infected patients (n = 291). Rifampin-resistant *M.tb* (n = 41), TB-IRIS (n = 51) and drug resistant bacterial infections (n = 12) were found in 12%, 14% and 3.4% of the 352 cases, respectively.

**Interpretation:**

In our setting, new AIDS defining illnesses, drug resistant *M.tb* and other drug resistant bacteria are important reasons for clinical deterioration in HIV-1 co-infected patients receiving antitubercular treatment. HIV-1 co-infected patients may be at increased risk of acquiring nosocomial drug resistant pathogens because profound immune suppression results in co-morbid illnesses that require prolonged inpatient admissions. Routine infection control is essential and needs to be strengthened in our setting.

## Introduction

In 2007, of an estimated total population of 47.9 million people in South Africa, an estimated 5.7 million were infected with HIV-1 and 0.35 million died from AIDS-related illnesses [1]. In 2006, the annual tuberculosis incidence rate was estimated to be 628 cases per 100,000 of the population per annum [2]. The two pandemics of tuberculosis and HIV-1 fuel each other. The annual incidence of tuberculosis disease doubles within the first year of HIV-1 infection[3] and may reach 30% per annum in the profoundly immune-suppressed [4]. Likewise, tuberculosis disease synergistically accelerates the progression of HIV-1 infection to Acquired Immune Deficiency Syndrome (AIDS) by inciting viral replication in immunologically activated CD4 cells [5], [6], [7]. In South Africa more than half of tuberculosis (TB) patients tested for HIV-1 are seropositive[8] and very many of the 22.5 million HIV-1 seropositive people residing in Sub-Saharan Africa are co-infected with *M. tuberculosis (M.tb)* [9].

Tuberculosis and HIV-1 control programmes in Africa are complicated by the increasing emergence of drug resistant *M.tb*, which can be associated with a very poor outcome and thus adversely affect overall tuberculosis control programme performance. In 2006, 44 HIV-1 seropositive patients died a median of 16 days after obtaining a specimen that cultured extensively drug resistant (XDR-) *M.tb* in a rural area of Kwa-Zulu Natal [10]. Nosocomial exogenous re-infection was implicated as two-thirds of patients were recently hospitalised before the diagnosis of XDR-tuberculosis and genotyping of isolates showed that 85% of patients were infected with a genetically similar isolate, belonging to the KZN family of strains. Focusing on patients worsening or not stabilising on antitubercular treatment is valuable in sentinel assessment of drug resistant *M.tb* transmission and mortality. Patients deteriorating on antitubercular treatment constitute a clinical subgroup in which one would predict higher rates of MDR- and XDR- *M.tb*. However, these patients may also deteriorate due to other reasons. Reasons for clinical deterioration[11] and death in patients on antitubercular treatment include co-morbid conditions [12], [13], [14], [15], exogenous re-infection or endogenous development of drug resistant *M.tb* [10], [16], tuberculosis associated-immune reconstitution inflammatory syndrome (TB-IRIS)[17], [18] and drug toxicities from treatment for tuberculosis, HIV-1 and/or concurrent opportunistic infections [19], [20], [21], [22]. Other causes are an incorrect diagnosis of tuberculosis, a paradoxical tuberculosis reaction (if not receiving antiretroviral therapy), poor adherence [23], incorrect antitubercular treatment, malabsorption of antitubercular treatment [24], as well as malabsorption of antibiotic drugs and subsequently altered pharmacokinetics (particularly in HIV-1 infected individuals with gastrointestinal problems). It is important to derive a rapid diagnostic work-up to determine cause of clinical deterioration as well as specific management to prevent further clinical deterioration and death.

This study aimed to determine which of these reasons for clinical deterioration on antitubercular treatment was most significant at an exceptionally busy urban district hospital situated in an area of high HIV-1 prevalence.

## Methods

### Setting

A prospective observational study conducted from 9 January to 8 April 2007 at GF Jooste Hospital (GFJH). GFJH is an urban 200-bed adult (>15 years age) district hospital in Cape Town, South Africa that serves approximately 1.3 million people and receives 8,000 referrals per month[25] from 30 primary care clinics.

The national tuberculosis programme manages new tuberculosis cases with 6 months treatment (isoniazid, rifampin, pyrazinamide, and ethambutol [HRZE] for 2 months followed by HR for 4 months [2HRZE/4HR]). The retreatment regimen adds streptomycin (S) as follows 2HRZES/1HRZE/5HRE. New tuberculosis cases do not routinely have tuberculosis drug susceptibility testing (DST) performed. Retreatment cases and patients not responding to antitubercular treatment may have DST performed.

Over 10,000 people have initiated combination antiretroviral treatment (cART) within the catchment area of GFJH (Meg Osler, Provincial Government of the Western Cape- personal communication). First-line cART in South Africa is stavudine (d4T), lamivudine (3TC) and either nevirapine or efavirenz (NVP, EFZ). EFZ is preferred in patients receiving rifampin-based antitubercular treatment. Patients with a CD4 count less than 200cells/mm3 and/or a history of a WHO stage 4 illness are eligible to commence cART [26]. In patients diagnosed with tuberculosis, cART is deferred if there is no history of a WHO Stage 4 illness and the CD4 count is greater than 200 cells/mm^3^. If there is a history of a WHO Stage 4 illness and/or the CD4 count is less than 200 cells/mm^3^ cART is commenced 2 months after initiating antitubercular treatment. If the CD4 count is less than 50 cells/mm^3^ or a serious HIV-1 related illness exists, cART can be commenced two weeks after initiating antitubercular treatment [26].

### Study procedures

#### Eligibility

Adult patients (>15 years) who received ≥14days antitubercular treatment within GFJH's catchment area and were referred by a medical doctor or nurse to the Emergency Department or Infectious Diseases Unit were assessed. Prior to referral, patients were diagnosed with tuberculosis at any of the 12 tuberculosis clinics within the catchment population of GF Jooste Hospital. If they deteriorated and were referred to GF Jooste Hospital they underwent renewed/repeated diagnostic procedures to either confirm tuberculosis disease (if previously microbiologically unconfirmed) and to exclude drug resistant *M.tb*. Patients were eligible if clinical worsening or failure to stabilise on therapy was confirmed on clinical assessment by a medical doctor at GFJH.

#### Tuberculosis diagnosis and referral

Microbiological confirmation of tuberculosis disease was defined as a specimen that cultured *M.tb* and/or was smear positive for acid-fast bacilli and that was obtained from a patient with symptoms and signs of tuberculosis [26]. An empiric diagnosis of tuberculosis was defined as follows: antitubercular treatment was initiated when the tuberculosis specimen was both smear negative for acid fast bacilli and culture negative or pending for *M.tb* but the South African National Tuberculosis Control Programme's case definitions for smear-negative and extra-pulmonary tuberculosis were fulfilled [26]. Patients commenced antitubercular treatment at their tuberculosis clinic and if they deteriorated or did not improve on treatment they were referred to our hospital for assessment.

#### Data collection

Clinical data regarding tuberculosis diagnosis, antitubercular treatment, HIV-1 status and antiretroviral therapy were recorded. Reasons for clinical deterioration were determined by clinical assessment and laboratory investigations performed by medical staff (doctors) working in the Emergency Department and Infectious Diseases Unit. All TB patients admitted to hospital were assessed by a specialist physician and reviewed weekly during inpatient stay by an Infectious Diseases physician. Investigations performed were according to clinical presentation and included C-reactive protein, full blood count (also called complete blood cell count), urea and electrolytes, serological and blood culture investigations. Specimens for tuberculosis microscopy, culture and sensitivity included sputum, pleural fluid, lymph node aspirates, ascitic fluid or cerebrospinal fluid (CSF). Chest radiography and computerised tomographic scanning were performed as indicated. Investigations also included sputum direct immuno-fluorescent antigen tests (DFAT) for *Pneumocystis jiroveci* pneumonia, CSF bacterial and fungal cultures and stool microscopy for coccidian parasites and culture for bacteria. All patients who develop drug-induced hepatitis are initially managed as inpatients according to a standardised protocol. The tuberculosis diagnosis is re-evaluated by reviewing results of all tuberculosis specimens. After clinical stabilisation and return to baseline of liver function tests, patients are carefully monitored and antitubercular drugs are sequentially rechallenged in cases of non-life threatening hepatitis and microbiologically proven tuberculosis. In severe cases (coagulopathy or encephalopathy), re-challenge of antitubercular treatment is not attempted. Instead, patients are treated with an alternative antitubercular regimen that does not involve rechallenge.

#### Tuberculosis diagnostics

The National Health Laboratory Services performed tuberculosis diagnostics. Ziehl-Nielsen staining was performed within 24 hours on all tuberculosis specimens, while auramine staining was performed on broth culture isolates. *M.tb* liquid culture, solid media susceptibility testing for isoniazid and rifampin, and quality assurance were performed as described elsewhere [27]. According to provincial protocol ethambutol (7.5 mcg/mL), ethionamide (20 mcg/ml), amikacin (30 mcg/mL), kanamycin (6 mcg/mL) and ofloxacin (2 mcg/mL) susceptibility testing was only performed in patients with rifampin resistance. Susceptibility testing to pyrazinamide, streptomycin and the remaining three second line drugs—terizidone, capreomycin, para-aminosalicylic acid— was not performed.

#### Definitions

Positive cultures for *M.tb* were categorised on the basis of drug susceptibility as: (i) susceptible to both isoniazid and rifampin; (ii) mono-resistant to rifampin; (iii) resistant to at least isoniazid and rifampin (MDR- *M.tb*); (iv) resistant to isoniazid, rifampin and either resistance to an injectable agent (amikacin or kanamycin) or ofloxacin (pre-XDR *M.tb*); and (v)resistant to isoniazid, rifampin, ofloxacin, and an injectable agent or ofloxacin (XDR *M.tb*) ‘Rifampin resistance’ was defined as any resistance to rifampin and included rifampin mono-resistant-, MDR- , XDR- and pre-XDR- *M.tb* [28].

Extended-spectrum beta lactamase (ESBL) producing bacteria were defined as bacteria having clavulanate-inhibited transferable enzymes able to hydrolyse third and fourth generation cephalosporins while methicillin resistant *Staphylococcus aureus* (MRSA) had an oxacillin minimum inhibitory concentration (MIC) ≥4 mg/l. TB-IRIS was defined according to a clinical case definition [29]. Adherence to antitubercular treatment was assessed by patient report, tuberculosis clinic cards (upon which daily doses taken are recorded) and/or collateral information from the health care worker at the tuberculosis clinic. No specific criteria were utilised but if no subsequent cause for deterioration was found and the patient had <80% adherence, the patient was assessed as having poor adherence. An additional illness to tuberculosis was defined as a second illness in patients where the initial tuberculosis diagnosis was confirmed by smear, culture and/or initial clinical response to antitubercular treatment where smear-negative and/or extrapulmonary tuberculosis was observed. It excluded poor adherence to antitubercular treatment, rifampin resistant *M.tb*, TB-IRIS or a paradoxical tuberculosis reaction. The use of concomitant cART distinguishes IRIS from a paradoxical TB reaction; IRIS occurs in patients receiving cART while paradoxical TB reaction occurs in the absence of cART. An alternate illness to tuberculosis was diagnosed when a clinical illness initially diagnosed as tuberculosis was not confirmed by smear or culture, there was no clinical response to antitubercular treatment and evidence of an alternate illness to explain the clinical presentation and course was found.

#### Outcomes

The primary outcome of the study was reason for clinical deterioration despite antitubercular treatment, with a specific focus on the proportion of rifampin resistant *M.tb*. The secondary outcome was outcome of admission.

#### Analysis

Data analysis was conducted using STATA-10 (Stata Corporation, College Station, Texas). Descriptive statistics were employed for basic characterization of variables. Wilcoxon rank-sum tests to compare medians, and Fisher's exact test of probability to compare proportions were used, as appropriate, to identify associations.

The Research Ethics Committee of the University of Cape Town approved the study (REF: 239/2007).

## Results

### Characterization of cohort

Three hundred and fifty-two patients met study inclusion criteria during the 3-month period. At initial tuberculosis diagnosis, 163 (46%) had pulmonary tuberculosis, 67 (19%) had extra-pulmonary tuberculosis and 122 (35%) had combined pulmonary and extrapulmonary tuberculosis. The median duration from initiation of antitubercular treatment to clinical presentation was 92.5 days (interquartile range, IQR 43–149). Eighty-four percent of TB patients (n = 296) required hospital admission for a median duration of 9.5 days (IQR 4–18). Admissions for patients deteriorating on antitubercular treatment accounted for 17% medical admissions (296/1,755) during the three-month study period.

**Figure 1:**
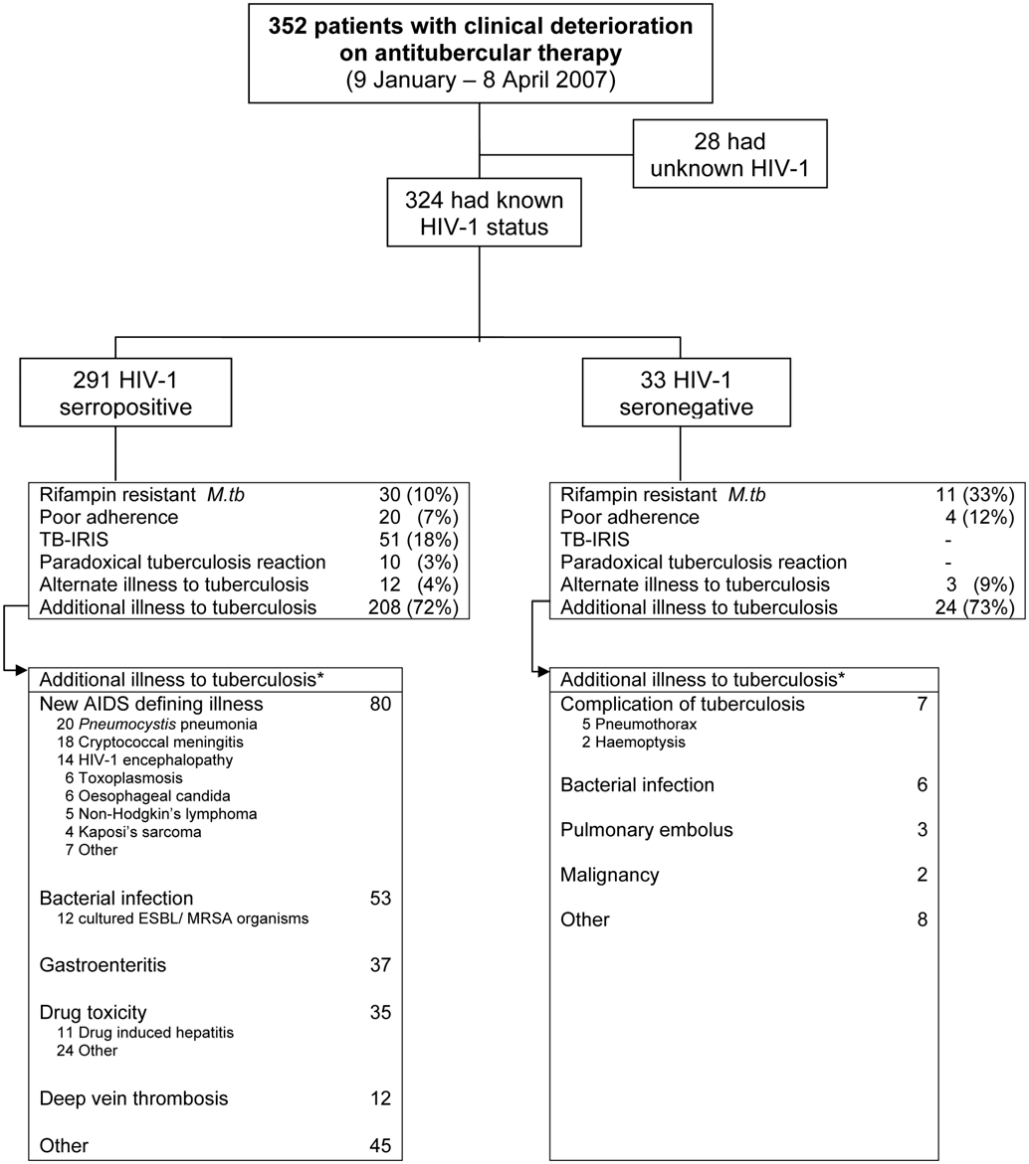
* Many patients had >1 cause for clinical deterioration, particularly additional illnesses. HIV-1 = human immunodeficiency virus, *M.tb = Mycobacterium tuberculosis*, TB-IRIS = tuberculosis associated-immune reconstitution inflammatory syndrome, Paradoxical tuberculosis reaction = initial clinical improvement with subsequent recurrence of tuberculosis clinical features but no evidence of drug resistant tuberculosis/or any other illness and patient not receiving antiretroviral therapy, MRSA = methicillin resistant *Staphylococcus Aureus*, ESBL = extended spectrum beta lactamase producing organism.

Eighty-three percent of TB patients (n = 291) were HIV-1 seropositive and 9% (n = 33) were HIV-1 seronegative (Figure 1). In 8% (n = 28), HIV-1 testing was not performed prior to referral or during investigation at GFJH; reasons included: not offered voluntary counselling and testing (VCT), VCT declined or too sick to obtain informed consent. Baseline features of TB patients stratified by HIV-1 status are shown in Table 1. HIV-1 seropositive TB patients were more frequently female, and less likely to have a past history of tuberculosis or microbiological confirmation (p≤0.01). The median CD4 count among HIV-1 seropositive TB patients was 89 cells/mm^3^ (n = 270). Of 255 patients who met the South African Department of Health criteria to receive cART [26], 122 (48%) were receiving this treatment.

**Table 1:** Characteristics of patients deteriorating on antitubercular treatment (N = 324) by HIV-1 status.

	HIV-1 +ve	HIV-1 −ve	P-value
	(n = 291)	(n = 33)	
**Demographics**			
Male – N (%)	138 (47.4)	24 (72.7)	0.006
Median age – years (range)	34 (16–86)	37 (19–68)	NS^1^
**Basis of tuberculosis diagnosis**			
Smear +ve – N (%)	106 (36.4)	22 (66.7)	0.001
Smear −ve/Culture +ve – N (%)	28 (9.6)	1 (3.0)	NS
Empiric diagnosis – N (%)^2^	157 (54.0)	10 (30.3)	0.01
**Previous tuberculosis**			
−n (%)	101 (34.7)	19 (57.6)	0.01
**Admitted**			
−n (%)	238 (81.8)	31 (94.0)	NS
Median duration – days (IQR^3^)	10 (4–18)	11 (5–22)	NS
**Outcome**			
Died as inpatient – N (%)	43 (14.8)	5 (15.2)	NS
**Illness contributory to death**			
Bacterial illness	12	1	
Enteric illness	8	-	
*Pneumocystis jiroveci* pneumonia	6	-	
Venous thromboembolism	5	2	
Drug side effects	3	-	
Rifampin resistant-tuberculosis^4^	3	-	
Kaposi sarcoma	2	-	
Cryptococcal meningitis	2	-	
Neurological TB-IRIS	2	-	
Chronic renal failure	-	1	
Acute coronary syndrome	-	1	

1 NS: not significant, p-value significant at p≤0.05.

2 i.e. No microbiological proof at commencement of tuberculosis treatment.

3 Interquartile range.

4 Rifampin resistant-tuberculosis = *Mycobacterium tuberculosis*, with resistance to at least rifampin, cultured from a patient with clinical deterioration of symptoms attributable to progressive tuberculosis disease.

### Final diagnoses

An additional illness to tuberculosis was detected in 72% of TB patients (n = 232) with known HIV-1 seropositive and HIV-1 seronegative status combined (n = 324) (Figure 1). Rifampin-resistant *M.tb* (n = 41), TB-IRIS (n = 51) and other drug resistant bacterial infections (n = 12) were diagnosed. Twenty-four TB patients had poor adherence to antitubercular treatment and fifteen patients had an alternate illness to tuberculosis.

#### Additional illness to tuberculosis

Additional illnesses to tuberculosis differed according to HIV-1 status (Figure 1). In the HIV-1 seropositive group (Table 2), new AIDS defining illnesses (n = 80), bacterial infection (n = 53), gastroenteritis (n = 37) and drug toxicity to cART, antitubercular, antibiotic or antineoplastic medications (n = 35) were most frequent. In the HIV-1 seronegative group, complications of tuberculosis, or its therapy (n = 7), and bacterial infection (n = 6) were most common.

**Table 2:** Organisms cultured and sites from which they were obtained*.

Organisms cultured	n	Site of cultured organisms	n
*Escheriae. coli*	17	Blood	13
*Klebsiella spp*	5	Urine	10
*Staphylococcus aureus*	8	Soft tissue abscess	9
*Proteus*	3	Sputum	3
*Acinetobacter*	3	Ascitic fluid	2
*Enterococcus*	2	Faeces	1
*Enterobacter*	1		
*Haemophilus influenza*	1		
*Streptococcus anginosus*	1		
*Streptococcus pneumonia*	1		
*Pseudomonas*	1		
*Salmonella type C*	1		

* 44 organisms were cultured from 38 sites from 35 patients: 3 patients cultured MRSA from ≥2 sites, 5 patients cultured 2 clinically significant bacteria from a single site.

#### Antitubercular drug resistance

Rifampin resistance *M.tb* was found in 41 TB patients. Eight had rifampin mono-resistant *M.tb*, 24 had MDR- *M.tb*, four had pre-XDR- *M.tb* and five had XDR- *M.tb*. Extended sensitivity testing was performed on only 15 (37%) of the 41 rifampin resistant tuberculosis cases; one third (5/15) of these patients had XDR- *M.tb*, while a further 27% (4/15) had pre-XDR- *M.tb*.

**Figure 2:**
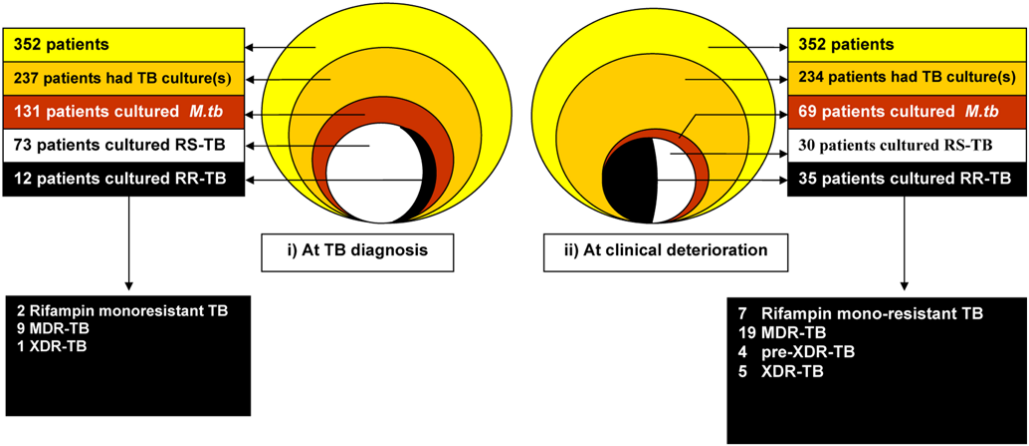
TB = Tuberculosis, RS-TB = rifampin sensitive *M. tuberculosis*, RR-TB = rifampin resistant *M. tuberculosis.* 6 of 12 patients who had rifampin resistant *M. tuberculosis* at initial tuberculosis diagnosis, re-cultured rifampin resistant *M. tuberculosis* at clinical deterioration. i.e. 29 new cases of rifampin resistant *M. tuberculosis* were diagnosed at clinical deterioration, 8 of 73 patients who had rifampin sensitive *M. tuberculosis* at initial tuberculosis diagnosis cultured rifampin resistant *M. tuberculosis* at clinical deterioration.

Figure 2 depicts TB culture results at initial TB diagnosis (left figure) and at subsequent deterioration (right figure). At initial tuberculosis diagnosis specimens were sent for tuberculosis culture in 237/352 (67%) patients, with 131 TB patients having a positive culture (Figure 2). Of 131 patients that cultured *M.tb* from specimens at initial tuberculosis diagnosis, 46 (35%) did not have drug susceptibility testing performed. Of the 85 TB patients who did have tuberculosis drug sensitivity analysis at diagnosis, 12 (14%) cultured rifampin resistant *M.tb* and 73 patients (86%) cultured rifampin sensitive *M.tb*.

At clinical deterioration specimens were sent for tuberculosis culture in 234/352 (66%) patients, with 69 patients culturing *M.tb*. Of these 69 patients, 4 (6%) did not have drug susceptibility testing performed. Of 65 patients who did have antitubercular drug sensitivity testing at clinical deterioration, 35 patients (54%) cultured rifampin resistant *M.tb* and 30 patients (46%) cultured rifampin sensitive *M.tb*.

Thus, at clinical deterioration, 29 new cases of rifampin resistant tuberculosis were diagnosed. These 29 cases had the following tuberculosis results at initial diagnosis: 6 *M.tb* sensitive to rifampin and isoniazid, 4 *M.tb* no sensitivities requested, and 3 no mycobacteria cultured. Sixteen of these 29 new cases of rifampin resistant tuberculosis had no tuberculosis culture sent at initial tuberculosis diagnosis.

Of 73 patients who had rifampin sensitive *M.tb* at initial tuberculosis diagnosis, eight (11%) cultured rifampin resistant *M.tb* at clinical deterioration. Of these eight, 2 patients cultured rifampin-mono-resistant *M.tb*, 5 MDR- *M.tb* and 1 XDR- *M.tb*, 12 to 419 days following the start of antitubercular treatment. Thirty seven percent of patients with rifampin resistant *M.tb* (15/41) are known to have died within six months of initial assessment at GFJH. The median duration from assessment to death was 28 days (IQR 11–72).

Forty-one patients who at initial TB diagnosis were culture negative (n = 11) or no culture was sent (n = 30), subsequently cultured *M.tb* at deterioration. In 11% (39/352) of cases, no tuberculosis cultures were sent at initial tuberculosis diagnosis and at clinical deterioration.

#### Extended spectrum beta lactamase producing organisms/methicillin resistant *Staphylococcus aureus*

At clinical deterioration, 214/352 (61%) TB patients had specimens sent for bacterial culture (128 of these TB patients had blood cultures performed). Of 214 TB patients that had ≥1 specimens sent for bacterial culture, 35 (16%) cultured clinically significant organisms. Cultured organisms and sites from which these specimens were obtained are detailed in table 2.

Of 53 HIV-1 seropositive TB patients treated empirically for bacterial infections (Figure 1), 32 had confirmatory cultures. Twelve of these TB patients cultured either extended spectrum beta lactamase (ESBL) producing organisms or methicillin resistant *Staph. aureus* (MRSA) (Table 3). The median CD4 count of these 12 TB patients was 57 cells/mm^3^ (IQR 21–80). Of nine TB patients culturing ESBL organisms, five had intermediate or high level resistance to amikacin while seven had high level resistance to ciprofloxacin. All were sensitive to imipenem, meropenem or piperacillin-tazobactam. Four TB patients had a septic illness due to MRSA, all were sensitive to vancomycin. One TB patient cultured both ESBL and MRSA organisms. Antibiotics administered to these 12 TB patients are shown in Table 3. Of these 13 drug-resistant isolates, nine (69%) were drug resistant bacteria cultured ≥48 hours after admission at GFJH (range 2–21days) while four (31%) were cultured on the day of admission. Six of the twelve (50%) TB patients were admitted to a hospital in the month preceding this GFJH admission for a median duration of 10 days (IQR 8–26). Nine TB patients (75%) with these infections died with the median time from obtaining the specimen to death being 10.5 days (IQR 6.3–14.5).

**Table 3:** Profile of patients with ESBL and MRSA organisms.

Case	Organism cultured	Site of specimen	Susceptibility to amikacin (A)/ciprofloxacin(C)/vancomycin (V)			Antibiotic received	CD4* (cells/mm^3^)	cART	Outcome of admission	Duration from obtaining specimen to death (days)	Specimen obtained >48 hrs after admission	Admitted to hospital in previous 30 days	Duration of previous hospital admission (days)
			A	C	V								
**1**	*Klebsiella spp.*(ESBL)	Blood	R	R	-	Ceftriaxone+ciprofloxacin	4	Yes	Died	5	Yes	No	-
**2**	*Klebsiella spp.*(ESBL)	Blood	I	R	-	-	89	No	Died	22	Yes	Yes	5
**3**	*Klebsiella spp.*(ESBL)	Blood	I	R	-	Ceftriaxone	77	Yes	Died	1	Yes	Yes	72
	*MRSA*	Pus swab ×2	-	-	S					13	No		
**4**	*E. coli* (ESBL)	Blood	I	R	-	Amikacin	20	Yes	Died	61	Yes	Yes	11
**5**	*E. coli* (ESBL)	Blood	S	R	-	Amikacin	51	No	Died	6	Yes	Yes	9
**6**	*E. coli* (ESBL)	Blood	S	R	-	Amikacin	76	No	Discharged alive	-	Yes	No	-
**7**	*E. coli* (ESBL)	Midstream urine	S	R	-	Amikacin	unknown	Yes	Discharged alive	-	Yes	No	-
**8**	*E. coli* (ESBL)	Blood	R	S	-	-	167	Yes	Died	10	No	Yes	30
**9**	*E. coli* (ESBL)	Blood	S	S	-	Amikacin	21	No	Died	11	No	No	-
**10**	*MRSA*	Pus swab ×2	-	-	S	Cloxacillin	38	No	Died	10	Yes	No	-
**11**	*MRSA*	Blood	-	-	S	Vancomycin	166	No	Died	8	Yes	No	-
**12**	*MRSA*	Pus swab ×2	-	-	S	Clindamycin	62	Yes	Discharged alive	-	No	Yes	7
**Median (IQR)**							**CD4 = 57 (21–80)**			**10 (8–14)**			
**Percentage**								**50%**	**Died 75%**		**Yes, 69%**	**Yes, 54%**	

*Klebsiella spp*. (ESBL) = *Klebsiella spp*. demonstrating extended spectrum beta-lactamase activity, sensitive to Imipenem, Meropenem or Piperacillin-tazobactam.

*E.coli* (ESBL) = *Escherichia coli* demonstrating extended spectrum beta-lactamase activity, sensitive to Imipenem, Meropenem or Piperacillin-tazobactam.

*MRSA* = Methicillin resistant *Staphylococcus aureus*, resistant to cloxacillin.

Susceptibility: R = Resistant, I = Intermediate resistance, S = sensitive.

cART = combination antiretroviral therapy.

* = all 12 patients were HIV-1-infected.

#### TB-IRIS

Fifty one (18%) of the 291 HIV-1 seropositive TB patients were diagnosed with TB-IRIS. Their median CD4 count nadir was 65 cells/mm^3^ (IQR 33–113). The median interval between initiation of tuberculosis therapy and initiation of cART was 69 days (IQR 35–94), and the median interval between cART initiation and onset of TB-IRIS symptoms was 14 days (IQR 7–24).

#### Alternate illness to tuberculosis

An alternate illness to tuberculosis was found in 9% of patients (16/181) who started empiric antitubercular treatment. In the HIV-1 infected group (n = 12) alternate illnesses were predominantly new AIDS defining illnesses including Kaposi's sarcoma (n = 2), *Pneumocystis jiroveci* pneumonia (n = 2) and non-Hodgkin's lymphoma (n = 2).

### Inpatient mortality

Inpatient mortality did not differ significantly according to HIV-1 status (p = 0.566, Table 1). Inpatient HIV-1 seropositive deaths (N = 43) were mainly due to bacterial infections (n = 12), new AIDS defining illnesses (n = 10), enteric illnesses (n = 8), and pulmonary embolism (n = 5).

An estimated 3500 patients start antitubercular treatment every three months at public sector primary care tuberculosis clinics within our catchment area (Judy Caldwell, Western Cape Tuberculosis Control Programme- personal communication). Private sector antitubercular treatment is not provided in our catchment area. During the 3-month study period 352 patients (10%, 95% confidence interval: 9–11%) deteriorated despite antitubercular treatment and required referral to our facility.

## Discussion

We undertook this study to investigate which reasons for clinical deterioration on antitubercular treatment were most significant in a resource-limited setting with a high prevalence of tuberculosis HIV-1 co-infection in Cape Town, South Africa. Focusing on this particular patient group may improve their outcomes and contribute to a rational use of limited resources especially as in our setting they account for 10% of patients started on antitubercular treatment.

We found that drug resistant *M.tb* and drug resistant bacterial infections were important reasons for clinical deterioration and death. Additional illnesses to tuberculosis accounted for most referrals, especially bacterial infections and new AIDS-defining illneses. Our findings are best explained in the context of rigorous admission criteria due to bed pressures at the 200-bed hospital; only 7.5% of adult patients referred to the emergency department are admitted to medical wards. Admission is prioritised for life-threatening illnesses requiring intravenous fluids or intravenous antibiotics (such as antibacterial or antifungal agents), drug resistant *M.tb* requiring daily inpatient intramuscular amikacin or kanamycin injections (while awaiting a bed at the nearby MDR- tuberculosis hospital) or patients requiring monitored supervision of antitubercular treatment because of severe disease. It is inevitable that a larger contingent of patients with clinical deterioration not meeting such strict admission criteria is not referred to hospital for assessment. A prospective study is needed to determine whether such a group exists, and if so whether the causes for clinical deterioration differ.

Nearly one-sixth of our patients cultured either drug resistant *M.tb* or other drug resistant bacterial infections. This has two important implications. Firstly we have identified a clinical subgroup of patients, namely patients deteriorating on antitubercular treatment, in whom the incidence of rifampin resistant tuberculosis (8.2%, 95% confidence interval: 5–11%) is high compared to the 2.5% of all tuberculosis cases reported to have MDR-tuberculosis in South Africa [30]. Secondly, tuberculosis HIV-1 co-infected patients in a hospital setting appear prone to acquire drug resistant bacteria such as ESBL and MRSA organisms. Studies need to be conducted at other health facilities to determine whether similar causes for clinical deterioration exist.

Nosocomial acquisition of XDR- *M.tb* among HIV-1 infected patients with a very poor outcome has recently been described in South Africa [10]; the contribution to death by co-morbid HIV-1 associated illnesses, however, was not discussed in that report. Our study suggests co-morbidities may play a role in the death of such patients.

The evolution from isoniazid and rifampin sensitive *M.tb* to rifampin mono-resistant-, MDR-, preXDR- *M.tb* and XDR- *M.tb* in eight patients may be due to initial mixed *M.tb* strain infection [31], exogenous re-infection with rifampin resistant *M.tb* [16] or the rapid development of *M.tb* drug resistance mutations despite a multidrug-regimen i.e. amplified drug resistance. Nosocomial re-infection of HIV-1 seropositive patients with MDR- and XDR- *M.tb* has been documented in both resource rich and resource constrained settings [10], [16]. The possibility that drug resistance evolved rapidly in drug susceptible cases receiving optimal treatment appears less likely based on clinical history and collateral information regarding adherence obtained from tuberculosis clinics and relatives. The decline in proportion of patients culturing *M.tb* may reflect either efficacious antitubercular treatment, inability of the patient to expectorate sputa for culture, or difficulty obtaining a non-pulmonary specimen for culture.

Nosocomial acquisition of drug resistant bacteria is also suggested by the temporal association between i) the timing of specimens that cultured ESBL and MRSA organisms and ii) the duration of current and previous hospital admissions. The unavailability of appropriate antimicrobial agents likely contributed to the high mortality rate in these 12 HIV-1 seropositive TB patients. At the time of the study, the standard regimen for suspected nosocomial sepsis was amikacin and ceftriaxone in the absence of renal impairment, and ciprofloxacin and ceftriaxone if present. Vancomycin was available to treat MRSA. Because of our study findings, ertapenem is now available.

The incidence of additional illnesses such as new AIDS defining illnesses, bacterial infections and gastroenteritis is indicative of the profound immune suppression in the HIV-1 infected group. Multiple opportunistic infections occur simultaneously in AIDS patients. A necropsy study of HIV-1 infected patients from Brazil reported more than one post-mortem diagnosis in 52% of the patients, and 48% had at least one AIDS-related disease not suspected clinically [32]. These researchers recommended aggressive investigation for infections and cancers in sick patients with AIDS, particularly in those not responding to initial antimicrobial therapy [32]. Bacterial infections and enteric illnesses were found in 26% and 18% of HIV-1 infected patients, respectively, in our study. Current provincial government protocols recommend starting co-trimoxazole prophylaxis in all HIV-1 positive patients one month after initiating antitubercular treatment in order to differentiate between side effects from antitubercular treatment and co-trimoxazole [26], [33]. This would likely reduce bacterial infections. Adherence to this recommendation was not assessed and needs to be determined in future studies. Although only 48% of patients eligible for cART were receiving cART at assessment for deterioration, it is likely that some patients subsequently initiated cART.

TB-IRIS was a final diagnosis in 18% (51/291) of HIV-1 seropositive patients. This probably reflects the high incidence of disseminated tuberculosis and the relatively late initiation of cART in profoundly immune suppressed HIV-1 patients in our setting. TB-IRIS is more likely to occur in patients with a low baseline CD4 count, a short duration between initiation of antitubercular treatment and cART and disseminated tuberculosis [34], [35], [36].

Fifteen cases of venous thrombo-embolic disease (12 deep vein thrombosis and 3 pulmonary embolus) were observed among this cohort. HIV-1 and rifampin are postulated risk factors for venous thrombo-embolic disease [37], [38].

Both HIV-1 uninfected and infected patients had prolonged admissions (9.5 days) compared to the typical duration of admission at GFJH (4 days) [25]. Longer inpatient admissions increase the risk of acquisition of nosocomial drug resistant pathogens, particularly in immune-compromised patients.

Our study's limitations relate fundamentally to its design within routine care in an exceptionally busy setting. Studies based in hospitals suffer referral bias and so the extent of the problem of clinical deterioration during antitubercular treatment cannot be precisely determined although is clearly very significant and likely to impact adversely on overall national tuberculosis programme success. The initial tuberculosis diagnosis was often defined by clinical algorithm rather than bacterial culture and, even at clinical deterioration, not all patients were sampled. Of all 352 TB patients assessed at deterioration, 182 (52%) did not culture *M.tb* at both tuberculosis diagnosis and at deterioration. Final diagnosis relied on available diagnostic modalities, better than in many parts of Africa but not state-of-the-art. Resistance to second line antitubercular agents was not always assayed: thus our estimates of pre-XDR and XDR- *M.tb* may be falsely low. 8% of patients were not tested for HIV-1 infection. Genotyping of drug resistance *M.tb* and other bacterial strains would have allowed us to better assess the likelihood of nosocomial transmission. All these factors have been considered in a clinic-based second study of this problem that is currently in progress.

As a result of these findings, basic infection control measures have been strengthened; extraction fans have been installed at high congestion areas and natural ventilation is encouraged to reduce *M.tb* transmission. N95 respirator masks are readily available to patients, relatives and health care workers to reduce aerosol transmission and infection of tuberculosis. Simple architectural modifications in the hospital are currently underway to further improve ventilation and reduce *M.tb* transmission risk.
